# Preface

**Published:** 1781-01

**Authors:** 


### PREFACE.

A Society of Phyficians who met together occafionally to converfe on medical fubjects, became defirous of enlarging the views of their inftitution, fo as to render their meetings of fome ufe to the public. For this purpofe they agreed to fet on foot a monthly publication, which fhould contain an account of new medical books and ufeful difcoveries in phyfic, and at the fame time be a repofitory for original effays. They were the more ready to engage in this undertaking, as a work upon fuch a plan feemed to have long been a defideratum in this country. One of their members, who was the firft and moft active promoter of this bufinefs, undertook the office of editor, to arrange the materials, fuperintend the printing of the work, &c. while others engaged to affift in the different branches for which they deemed themfelves beft qualified. Proper arrangements were made for obtaining the neceffary fupply of foreign medical publications; and that no means might be omitted for procuring early intelligence of medical news, a correfpondence was eftablifhed in different parts of the Continent.

The Editors have now completed their firft volume; and have the fatisfaction to find that their work has met with a favourable reception from the public. This circumſtance, as it affords them the ſtrongeſt preſumptive proof of the utility of their labours, cannot fail of inducing them to proceed; and their numerous readers may be aſſured, that as they engaged in this undertaking on the moſt liberal principles, ſo they will continue to proſecute it with the utmoſt candour, and with unremitting attention.

The value of that part of their work which is intended for Eſſays and Obſervations, muſt depend in a great meaſure on the contributions of communicative practitioners. Hitherto they have been extremely fortunate in this department; the original papers with which they have been enabled to enrich their firſt volume, being ſuch as would do honour to any collection. The Editors, deſirous of rendering this part of their Journal every day more and more intereſting, earneſtly intreat Gentlemen of the Medical Profeſſion in every part of the world to contribute towards it, by communicating to the Society accounts of uſeful caſes, epidemical diſeaſes, and ſuch other matters as may tend to the improvement of medical ſcience.

